# Administration of darunavir tablets in patients with difficulties in swallowing – two case reports

**DOI:** 10.1186/1758-2652-13-S4-P114

**Published:** 2010-11-08

**Authors:** S Scholten, S Mauruschat, S Hindermann, B Ranneberg

**Affiliations:** 1 Praxis Hohenstaufenring, Cologne, Germany; 2 private practice, Wuppertal, Germany; 3 Janssen, Johnson&Johnson-Platz 1, Neuss, Germany

## Background

For various reasons it can be necessary to administer antiretrovirals permanently or temporarily in a dissolved form: swallowing difficulties, stomach tube or patients in an ICU. However, not all drugs are available as oral solution. Crushing and dissolving may be an option, but data on plasma levels and efficacy are limited or not existing.

## Methods

We report efficacy and plasma level data for two patients, one with dysphagia and Candida esophagitis and one with a stomach tube, who received darunavir (DRV) crushed and dissolved.

## Results

Patient 1 is 57 year old HIV+ male (CDC C3), first diagnosed in 1990, who has been on various antiretroviral regimens since 1992. He presented with a VL of 72.551 copies/mL, 56 CD4 cells/mm^3^ and candida esophagitis. HAART was initiated with DRV/RTV 600/100 mg in combination with etravirine (ETR) and raltegravir (RAL). DRV and RAL tablets were crushed, ETR was suspended and ritonavir (RTV) was given as oral solution. The medication was well accepted with exception of RTV oral solution. Trough levels (10 h post dose) for DRV were 6.950 ng/mL; measured one month post treatment initiation. At that time, VL had declined to 102 copies/mL; CD4 count had increased to 111 cells/mm^3^. The patient was then switched to tablets, viral load has been <40 copies/mL since then.

Patient 2 is a 48 year old HIV+ paraplegic woman (CDC C3), first diagnosed in 2004 with a permanent stomach tube. HAART with DRV/RTV 600/100 mg bid, RAL and tenofovir/emtricitabine via tube was initiated in 2008. Since then, viral load has been permanently < 40 copies/mL; CD4 count is stable between 440 and 540 cells/mm^3^. Plasma levels for DRV were within the therapeutic range: 4.430 ng/mL (5 hours post dose) in January and 5.210 ng/mL (3 hours post dose) in June.

## Conclusions

Crushing DRV tablets and combining them with RTV oral solution reached adequate DRV plasma levels. In these two patients, HAART administered via artificial feeding was an effective option for short and long-term treatment.

